# Apoptosis induction by combination of drugs or a conjugated molecule associating non-steroidal anti-inflammatory and nitric oxide donor effects in medullary thyroid cancer models: implication of the tumor suppressor p73

**DOI:** 10.1186/s13044-015-0025-3

**Published:** 2015-08-14

**Authors:** Thierry Ragot, Claire Provost, Aurélie Prignon, Régis Cohen, Michel Lepoivre, Sylvie Lausson

**Affiliations:** UMR 8203, Gustave Roussy, Laboratoire de Vectorologie et de Thérapeutiques Anticancéreuses, Villejuif, 94805 France; UMR 8203, CNRS, Laboratoire de Vectorologie et Thérapeutiques Anticancéreuses, Villejuif, 94805 France; UMR 8203, Univ Paris-Sud, Laboratoire de Vectorologie et Thérapeutiques Anticancéreuses, Villejuif, 94805 France; Sorbonne Universités, UPMC University Paris 06, plateforme LIMP, Laboratoire d’Imagerie Médicale Positonique, Hôpital Tenon, Paris, 75020 France; Hopital Delafontaine, Endocrinology Unit, Saint Denis, France; IBBMC, CNRS 8619, bat 430, Université Paris Sud XI, Orsay, Paris, 91405 France

**Keywords:** Medullary thyroid carcinoma, Non-steroidal anti-inflammatory drugs, NO-donors, Apoptosis, TT cells, rMTC 6–23 cells

## Abstract

**Background:**

Medullary thyroid cancer (MTC) is a C-cell neoplasm. Surgery remains its main treatment. Promising therapies based on tyrosine kinase inhibitors demand careful patient selection. We previously observed that two non-steroidal anti-inflammatory drugs (NSAID), indomethacin, celecoxib, and nitric oxide (NO) prevented tumor growth in a model of human MTC cell line (TT) in *nude* mice.

**Methods:**

In the present study, we tested the NO donor: glyceryl trinitrate (GTN), at pharmacological dose, alone and in combination with each of the two NSAIDs on TT cells. We also assessed the anti-proliferative potential of NO-indomethacin, an indomethacin molecule chemically conjugated with a NO moiety (NCX 530, Nicox SA) on TT cells and indomethacin/GTN association in rMTC 6–23 cells. The anti-tumoral action of the combined sc. injections of GTN with oral delivery of indomethacin was also studied on subcutaneous TT tumors in *nude* mice. Apoptosis mechanisms were assessed by expression of caspase-3, TAp73α, TAp73α inhibition by siRNA or Annexin V externalisation.

**Results:**

The two NSAIDs and GTN reduced mitotic activity in TT cells versus control (cell number and PCNA protein expression). The combined treatments amplified the anti-tumor effect of single agents in the two tested cell lines and promoted cell death. Moreover, indomethacin/GTN association stopped the growth of established TT tumors in *nude* mice. We observed a significant cleavage of full length PARP, a caspase-3 substrate. The cell death appearance was correlated with a two-fold increase in TAp73α expression, with inhibition of apoptosis after TAp73α siRNA addition, demonstrating its crucial role in apoptosis.

**Conclusion:**

Association of NO with NSAID exhibited amplified anti-tumoral effects on *in vitro* and *in vivo* MTC models by inducing p73-dependent apoptotic cell death.

## Introduction

Medullary thyroid carcinoma (MTC) is a neuroendocrine neoplasm of C cells (for reviews see [1, 2]). This cancer releases large amounts of calcitonin (CT) correlated to tumor size [3]. MTC is sporadic in about 75 % of cases, and patients with sporadic carcinoma are usually diagnosed at late stage. Nowadays, surgical removal of the thyroid with lymph node dissection is the gold standard curative treatment of MTC. Prognostic remains poor since 40 to 60 % of patients are not cured. Current chemo- and radiotherapies are still ineffective. Hereditary germ-line mutations or somatic mutations of the RET proto-oncogene are involved in the carcinogenesis of familial and sporadic MTCs respectively. Several tyrosine kinase inhibitors (TKIs) that are notably RET receptor inhibitors are used and under evaluation for patients with advanced MTC with promising results [2, 4]. Thus TKIs vandetanib or cabozantinib can be used as single agent for first line systemic therapy in selected patients with advanced progressive MTC. But TKIs give only a significant increase of progression-free survival with no effect on death rate [5]. Thus, other or combined curative approaches are necessary to improve MTC treatments in the future.

The anti-tumor potential of non-steroidal anti-inflammatory drugs (NSAIDs) was recognized a few years ago [6–8]. The anti-proliferative effects of NSAIDs can result from the decrease of prostaglandin (PG) synthesis by inhibition of cyclooxygenase (COX) activity but also from anti-tumoral actions independent of PGs and COXs. We have previously demonstrated that the classical NSAID, indomethacin, reduced the development of xenografted TT tumors induced by injection of human MTC TT cells in *nude* mice [9]; indomethacin lowered PGE_2_ secretion by TT cells. We also reported that a low dose of the selective COX-2 inhibitor, celecoxib (with less gastro-intestinal side effects than conventional drugs) significantly diminished the growth of TT tumors. This anti-tumor action was independent of PGE_2_ and COX-2 [10].

Otherwise, we observed a very strong anti-tumor potential of nitric oxide (NO) on MTCs for two rat cell lines and TT cells [11]. More recently, authors reported that the chemical association of NO donors with a NSAID not only prevented side effects of the NSAID but also was able to amplify its anti-tumor action [12–14]. Elevation of the NO concentration can cause DNA damage, mutation and apoptosis [15]. The tumor suppressor protein p53 is a key player in the DNA damage response and the onset of apoptosis. NO has been shown to activate p53 which promotes pro-apoptotic effects [16]. Two *p53* related genes, *p63* and *p73*, have also been identified more than 15 years after the discovery of *p53* [17, 18]. The human *p*73 gene generates two groups of isoforms, some with a complete transactivation (TA) domain (TAp73) and others exhibiting a truncated TA domain (ΔNp73). Growth suppression or induction of apoptosis can be accomplished by the TAp73 isoforms. Studies have shown that p73 is required for apoptosis induction in response to DNA damage by chemotherapeutic drugs such as cis-platin [19]. Thus, p53 and p73 could be interesting targets to study in MTC chemotherapies: *p53* is not mutated in this cancer; in contrast, p73 was never studied in C cell but mutations in the *p*73 gene are rare in cancer patients. p73 is not only involved in tumor suppression but has important functions in neural cells and it also could be well expressed in neuroendocrine C cells.

In the present study, we compared the anti-proliferative actions of a pharmacological dose of the NO-donor, glyceryl trinitrate (GTN) or Trinitrine, a drug used in cardiology, of two NSAID, indomethacin and celecoxib, of the combinations of one NSAID with GTN, and finally, of a chemically conjugated molecule, NO-indomethacin (NCX 530) from Nicox SA, an indomethacin molecule conjugated with a NO donor group. We also studied the implications of cell death mediators p53 and p73 in TT cells. The NSAID doses used in these cultures were those reproducing the anti-tumoral benefits with low side effects we have previously described *in vivo*. Moreover, we assessed the anti-proliferative effect of the indomethecin/GTN association in another MTC cell line, rMTC 6–23 and studied the anti-tumoral action of a combined administration of GTN in sc. injections with oral delivery of indomethacin on xenografted TT tumors.

## Materials and methods

### Materials

Celecoxib was a generous gift from Pfizer (USA). Indomethacin was purchased from Cayman Chemical (Ann Arbor, MI). The NO-donor glyceryl trinitrate (GTN) was obtained from Merck (Lyon, France). The conjugated molecule NO-indomethacin (NCX 530) was a gift from Nicox SA (Sophia Antipolis, France). The TT and rMTC 6–23 cell lines were from the American Type Culture Collection (Rockville, MD). Primary antibodies to p73 (IMG 313A, Imgenex, A300-126A, Bethyl Laboratories), p53 (sc 6243, Santa Cruz Biotechnology), PCNA (Santa Cruz Biotechnology), PARP-1 (c-2-10, Calbiochem) and α-tubulin (T9026, Sigma) were used for western blots. Detections were performed with fluorescent Alexa Fluor 680-conjugated anti-mouse (A 21057, Invitrogen/Molecular Probes) or IRDye 800CW-anti-rabbit (926–32211, LI-COR Biosciences) IgG. Rabbit anti-caspase-3 antibody (ab52293, Abcam), Starr Trek Universal detection kit (Biocare Medical/Eurobio) and AEC Peroxidase Substrate kit (Vector Laboratories/Eurobio) were used for the immunohistochemitry of TT tumor slides.

### Cell culture

TT cells were cultured in RPMI 1640 medium (Invitrogen, Cergy-Pontoise, France) supplemented with 2 mM L-glutamine, 25 mM HEPES, 10 % heat-inactivated fetal calf serum (FCS), 100 U/mL penicillin and 100 μg/mL streptomycin (Invitrogen). RMTC 6–23 cells were cultured in Dulbecco’s Modified Eagle’s medium (DMEM) GlutaMAX™ (Life Technologies, Cergy-Pontoise, France) supplemented with 1 % of Non-Essential Amino Acids (Life Technologies) and −5 % heat-inactivated FCS. To determine the anti-proliferative effects of the NSAID/GTN combinations or NCX 530, TT or rMTC 6–23 cells were seeded out in 6-well-plates at a density of 2 to 3 × 10^5^ cells per well, respectively. Three days later, NSAIDs and/or GTN were added (or not, or vehicles only, for the controls) to cell culture medium. Medium was changed every two days (TT cells) or every 36 h (rMTC 6–23 cells). After various periods of treatment, cells were dissociated 5 min with trypsin-EDTA or TrypLE™ Express (Life Technologies) for TT or rMTC 6–23 cells, respectively. Cells were counted with an hemocytometer (Trypan Blue exclusion) and/or using counting slides with an automated cell counter (TC20™ Bio-Rad, Marnes-la-Coquette, France). NSAIDs were dissolved in DMSO and GTN in ethanol. The following doses were used: celecoxib 25 μM, indomethacin 100 or 200 μM, GTN 100 μM, and NCX 530, 100 and 200 μM for short term experiments, and 150 μM for long term experiments. Controls were performed in the presence of drug vehicules (DMSO and ethanol) at the same concentrations as in experimental wells (DMSO ≤ 0.4 % and ethanol = 0.2 %). For western blot analyses, cells were seeded out in 6-well plates at a density of 10^6^ cells per well. Treatments began 3 days later. In proliferation and apoptosis detection experiments, triplicates were performed for each category; in experiments for immunoblotting analyses, duplicates were used.

### Apoptosis detection

After cell counts, the cell suspensions were adjusted to 10^6^ cells/ml in microfuge tubes. An Annexin V-FITC Apoptosis Detection kit (Calbiochem, Merck Millipore, Darmstadt, Germany), was used to quantify apoptosis/necrosis. The “Rapid Annexin V binding” protocol provided by the suppliers was followed. After incubation with Annexin V-FITC and addition of Propidium Iodide, fluorescence was immediately analyzed by flow cytometry (BD Accuri™ C6 Flow Cytometer, BD Biosciences) using 530/30 and 670 LP emission filters preventing spillover. Unlabeled samples were used to adjust the gates.

### Immunoblotting analyses

Immunoblots were performed as described in Tebbi et al. [20]. After harvest, cells were washed twice in ice-cold phosphate buffered saline (PBS). Crude cell extracts were prepared in a 50 mM Tris–HCl lysis buffer, pH 7.4, supplemented with 150 mM NaCl, 1 % Triton X-100, 0.5 % sodium deoxycholate, 1 mM EDTA, 1 mM DTT and protease inhibitors including 1 mM Pefablock (Merck/Calbiochem). Soluble proteins (30 μg per lane) were separated by SDS-PAGE using a 10 %-acrylamide gel and transferred onto nitrocellulose membranes. These ones were blocked in 3 % skimmed milk. After overnight incubation at 4 °C with primary antibodies and four washes in PBS/Tween, membranes were incubated for 1 h with fluorescent dye-conjugated secondary antibodies. After washing, the infrared fluorescent signals at 680 and 800 nm were quantified with an Odyssey scanner (LI-COR Biosciences). Protein contents were standardized using α-tubulin band density.

### Transfections with TAp73 siRNA

SiRNA sequences were reported and validated in Guittet et al. [21]. Cells were transfected with TAp73 Select siRNA (Ambion) or control siRNA (MWG) using Interferin transfection reagent (Polyplus Transfection). TT cells were plated in 6-well plates and grown to approximately 30-50 % confluency. Before transfection, culture medium was replaced by the same medium without antibiotics (4 mL per well). Interferin (corresponding to 15 μL per well) was incubated with siRNA for 10 min in antibiotic-free medium. Then, the mixture was added dropwise to wells, resulting in a siRNA concentration of 30 nM. We have previously verified that this method led to 80 % reduction of TAp73α expression, 3 days after mixture addition. Cells were maintained in the transfection medium for 16 h. They were then cultured in usual medium with GTN (100 μM) and celecoxib (25 μM) treatment or with DMSO and ethanol (control wells) during 2 days and 8 h.

### *In vivo* experiment

5 × 10^6^ TT cells were inoculated subcutaneously (sc) on the back of each *nude* mouse. When tumors became palpable, their diameters were regularly measured with a caliper and tumor volume (mm^3^) was calculated using the following formula: (the shortest diameter)^2^ × (the longest diameter) × 0.5. When the mean volume of tumors reached about 70 mm^3^, animals were randomly divided into three groups: one control group (5) and two treated groups, one receiving sc injections of Nitronal each two days, (0.1 mg GTN/20 g of body weight during 6 days, then 0.15 mg, *n* = 5), the other group receiving the same doses of Nitronal with indomethacin (2 mg/day/kg of body weight in drinking water, *n* = 4). Animal manipulations were performed according to the recommendations of the French Ethical Committee and under the supervision of authorized investigators.

Mice were sacrificed at day 14 and tumors were removed and weighted. Tumors were divided in two parts. One part of the tumor was fixed in 4 % paraformaldehyde during 96 h and embedded in paraffin. Some tumor slides, chosen in three different levels (100 μm apart), were colored with hematotoxylin-phloxine-saffron (HPS) stain. The cavity area of colored slides was measured by ImageJ software at three levels in each tumor. For caspase-3 immunohistochemistry, we followed a method similar to that described in Bressenot et al. [22].

### Statistical analyses

All results were analysed using ANOVA. Differences between two means were tested by the Fisher tests. Data are represented as means ± SEM. For each determination, two or three independent experiments were performed. The significance level was set at *P* < 0.05. For the proliferation experiments, we assessed the differences between treated cells and control cells at day 4 (D4). We also tested cell number reductions induced by NO-NSAID bi-therapies at D4 and D8 versus D0. For the immunoblotting analyses, we tested the differences between treated and control cells at D1 and D3. Moreover, for each NSAID/GTN combination (proliferation experiments at D4 and p73 expression at D3), we performed a two-factor variance analysis to assess the significance of NSAID or GTN effects individually and to test the presence of a NSAID/GTN interaction.

In the annexin V studies, a significant linear regression was obtained between apoptotic cell percentages and total number of cells, in controls: the apoptotic cell percentage increases when cell density grows. Thus, we compared the apoptotic cell percentage in each treated culture with a percentage (calculated with the regression) in a control containing the same total number of cells than the treated culture. For the *in vivo* experiment, we assessed the tumor volume growth and the mean differences between treatments by the two-factor analysis (time factor as repeated measures and treatment factor).

## Results

### Anti-proliferative effects of GTN, NSAID/GTN combinations and NO-indomethacin on TT cells

**Fig. 1:**
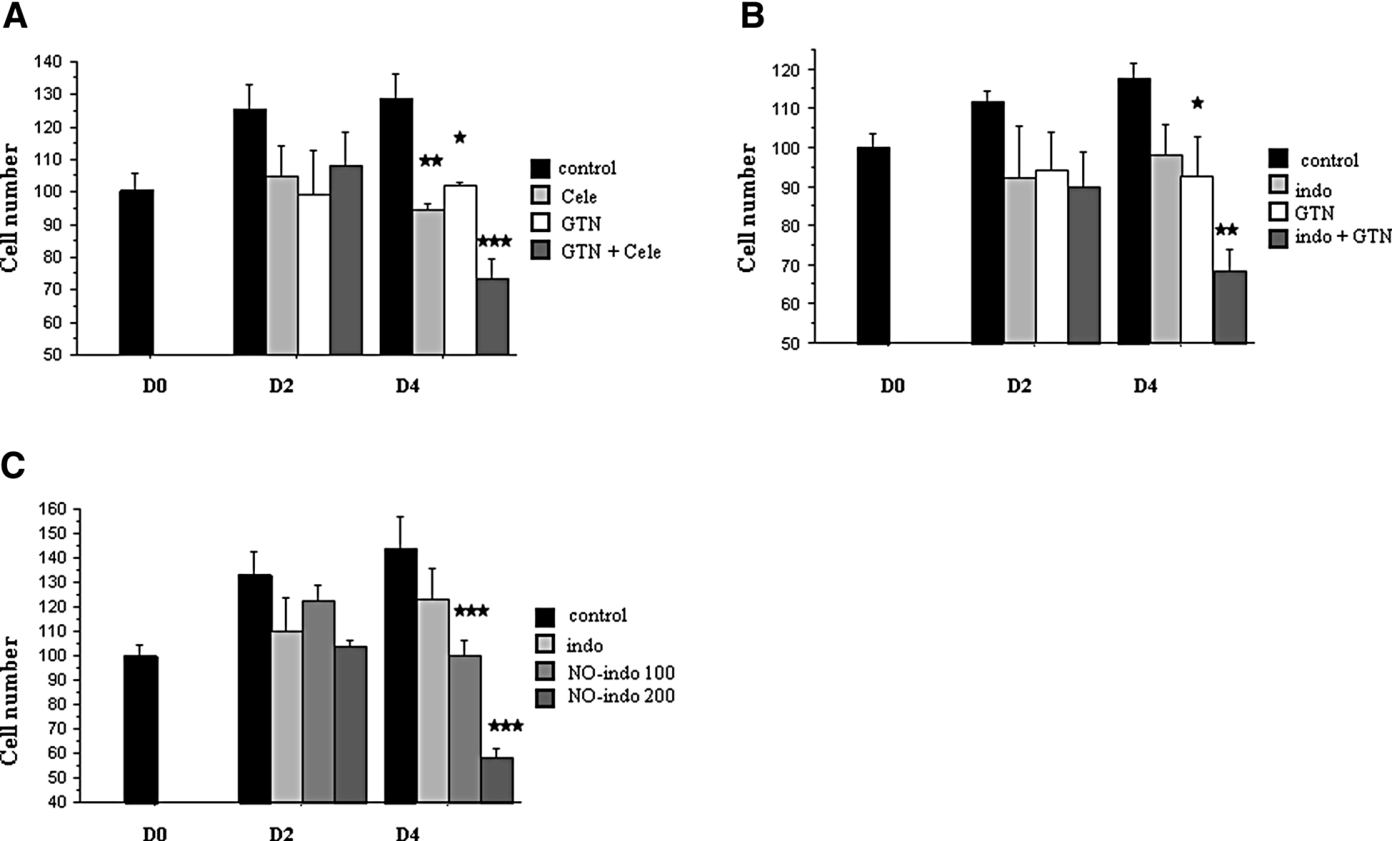
**a** Evolution of the number of viable, control or treated TT cells: 25 μM celecoxib, (cele), 100 μM GTN or a combination of celecoxib plus GTN, during 4 days. **b** Evolution of viable TT cell number during 4-day treatments with 100 μM indomethacin (indo), 100 μM GTN or a bi-therapy of indomethacin plus GTN. **c** Effects of the conjugated molecule (NO-donor conjugated to indomethacin, NCX 530), 100 μM (NO-indo100) or 200 μM NO-indomethacin (NO-indo200), during the first 4 days of treatment. Representative graphs of three (**a**, **b**) or two (**c**) independent experiments performed in triplicates. Adherent cells were dissociated with trypsin-EDTA and count with an hemocytometer with blue trypan exclusion. * = *P* < 0.05, ** = *P* < 0.01, *** = *P* < 0.001 versus control (Fisher test)

As previously reported, we observed that the NSAIDs celecoxib and indomethacin prevented the proliferation of TT cells: the cell number in treated samples was reduced by about 30 % at D2 and D4 versus controls (Fig. 1a and b). Moreover, we established that a NO-donor, GTN (100 μM), had a similar effect: the viable cell number was decreased by about 30 % versus controls (Fig. 1a and b). We revealed significant effects of each NSAID alone and GTN alone at D4. Interestingly, the combinations of one NSAID with GTN strongly increased the anti-tumoral action seen for each individual molecule at D4 (cell number diminution of 50 to 60 % versus controls). Significant reductions were observed between D0 and D4, *P* < 0.05, after celecoxib/GTN incubation and *P* < 0.01, after indomethacin-GTN treatment. The two-factor analysis of variance did not reveal an interaction between each NSAID and GTN; this result suggests the presence of additive effects. NO-indomethacin, NCX530, had the same anti-tumor action than the GTN and indomethacin incubation. The reduction of cell population treated with NCX 530 versus control was dose-dependent (Fig. 1c).

### Continuous anti-tumor action of NSAID/GTN combinations and NO-indomethacin after long-time administration on TT cells

**Fig. 2:**
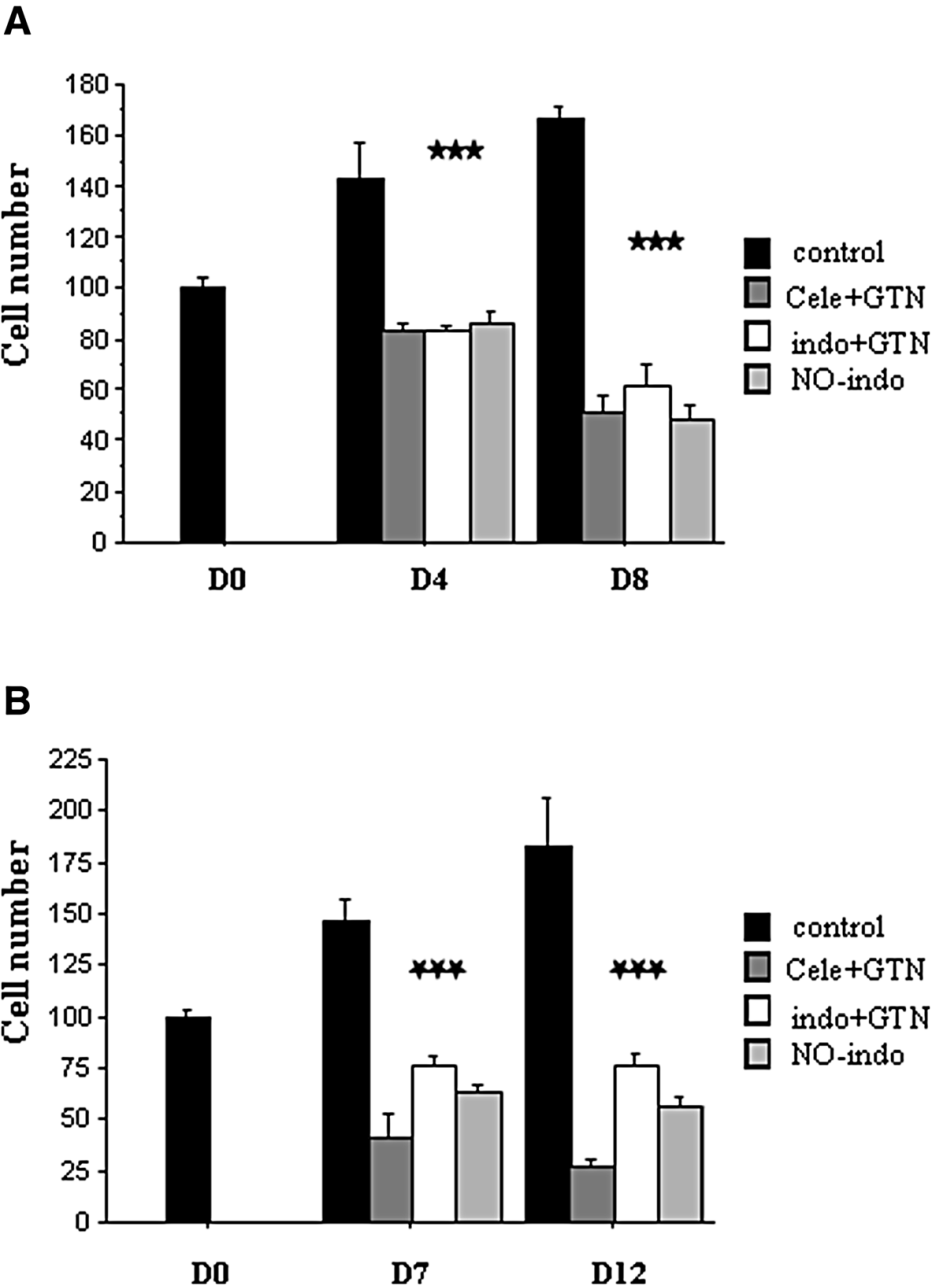
**a** Evolution of viable TT cell number in control wells and in wells treated by NSAID/GTN combination: 25 μM celecoxib plus 100 μM GTN (cele + GTN), or 100 μM indomethacin plus 100 μM GTN (indo + GTN), or 150 μM NCX 530 (NO-indo), during 8 days. **b** Evolution of viable TT cell populations after cessation of treatments with NSAID/GTN bi-therapies or NCX 530 (25 μM celecoxib, 100 μM indomethacin, 100 μM GTN or 150 μM NCX 530). Representative graphs of two independent experiments performed in triplicates. Adherent cells were dissociated with trypsin-EDTA and count with a hemocytometer. *** = *P* < 0.001 versus control for each treatment (Fisher tests)

When cells were treated with NO-NSAID combinations until D8, the cell number reduction was prolonged from D4 to D8: *P* < 0.05 for indomethacin/GTN, *P* < 0.01 for celecoxib/GTN and NO-indomethacin alone (Fig. 2a). In another experiment, TT cells were incubated with NSAID/GTN combinations or NO-indomethacin during 7 days then, cultured in drug-free medium until D12. Under these conditions, no growth rebound was observed between D7 to D12 (Fig. 2b). Thus, the anti-tumoral action was amplified when treatments were prolonged and this beneficial effect was maintained after treatment cessation.

### NSAIDs, GTN and NSAID/GTN bi-therapies reduce mitotic proliferation of TT cells. NSAID/GTN combined treatments only lead to cell death

**Figure 3:**
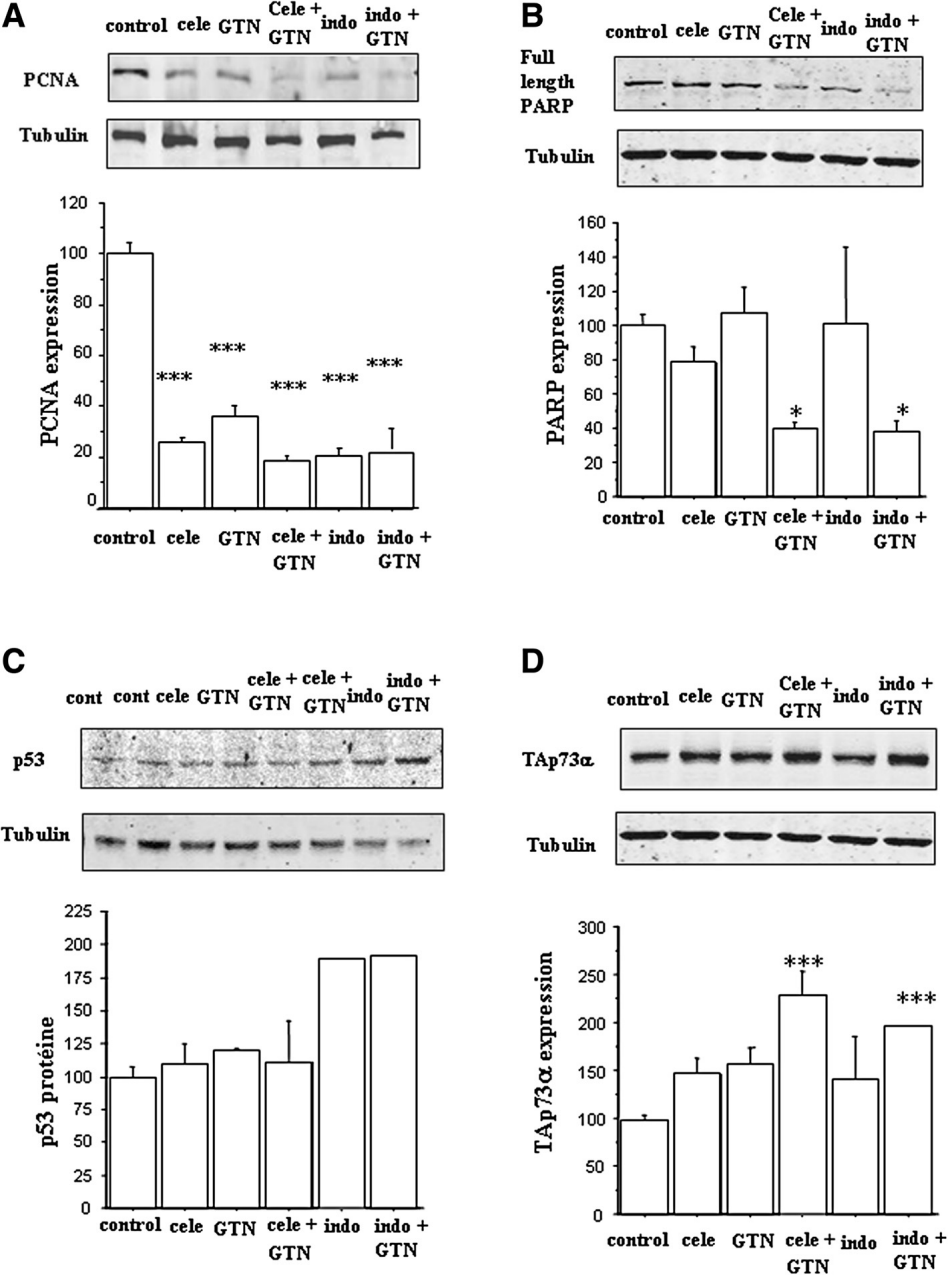
Expression of various proteins in TT cells after 3 days of exposition to 25 μM celecoxib (cele), 100 μM indomethacin (indo), 100 μM GTN or NSAID/GTN combinations. **a** PCNA protein. For each category, *n* = 6 to 3. **b** PARP heavy chain. For each category, *n* = 4 to 3. **c** Tumor suppressor p53. For each category, *n* = 4 to 2. **d** TAp73α. For each category, *n* = 7 to 4. Graphs obtained from associated results of two experiments on TT cells. Proteins from TT cell lysates were separated by SDS-PAGE using 10 % acrylamide gel and transferred onto nitro cellulose membranes. Immunoblots were probed with specific antibodies and a α-tubulin (internal control) antibody. * = *P* < 0.05, *** = *P* < 0.001 versus control (Fisher tests)

PCNA is a nuclear antigen expressed during cell mitotic division. The effects of drug exposition on PCNA expression were investigated by western blot analysis. Figure 3a shows a significant decrease in PCNA levels in all 3-day-treated cells versus controls: celecoxib alone, *P* < 0.0001, indomethacin alone, *P* < 0.0001, GTN alone, *P* < 0.0001, NSAID/GTN combinations, *P* < 0.0001 (NCX 530, *P* < 0.01). Thus, all these treatments reduced the levels of TT cell mitotic divisions.

PARP is a substrate of caspase-3, cleaved early during cell apoptosis. Western blot analyses revealed that PARP was cleaved in TT cells incubated with drug combinations (or NO-indomethacin alone, data not shown) during 3 days: the levels of PARP full-length polypeptide were significantly decreased (*P* < 0.05 versus control cells) for all bi-therapies, (Fig. 3b). No effect appeared after one day of treatment (data not shown). Thus, the reduction of viable cell numbers receiving bi-therapies after D2 also resulted from apoptotic cell death induction.

### Increased expression of the tumor suppressors p53 and p73

We investigated the expression of the tumor suppressors p53 and p73 in TT cells at basal levels and in response to the different treatments by NSAIDs and NO. We found that this cell line expressed p53 and TAp73α at basal levels. ΔNp73 was not detected in control and treated TT cells. No change in p53 and p73 expressions was observed in 1-day treated cells (data not shown). Indomethacin, indomethacin plus GTN, and NO-indomethacin led to comparable increases in p53 levels at D3 while celecoxib and GTN had no effect (Fig. 3c).

In contrast, TAp73α expression increased by a factor two after 3-day NSAID/GTN combined treatments (Fig. 3d) or incubation with NO-indomethacin alone. These elevations were correlated with a reduction in PARP levels and TT cell number decrease between D2 and D4. The two-factor analysis of variance showed significant 50 %-increases of TAp73 level after 3-day treatments with NSAID alone or GTN alone: in the experiments with indomethacin plus GTN, *P* = 0.05 for indomethacin factor, *P* = 0.01 for GTN factor; in the experiment with celecoxib plus GTN, *P* < 0.05 for celecoxib and for GTN. No significant interaction between NSAIDs and GTN was revealed. Thus, additive effects lead to the strongest increases in TAp73 expression after 3-day combined treatments.

### p73 siRNA transfection

**Fig. 4:**
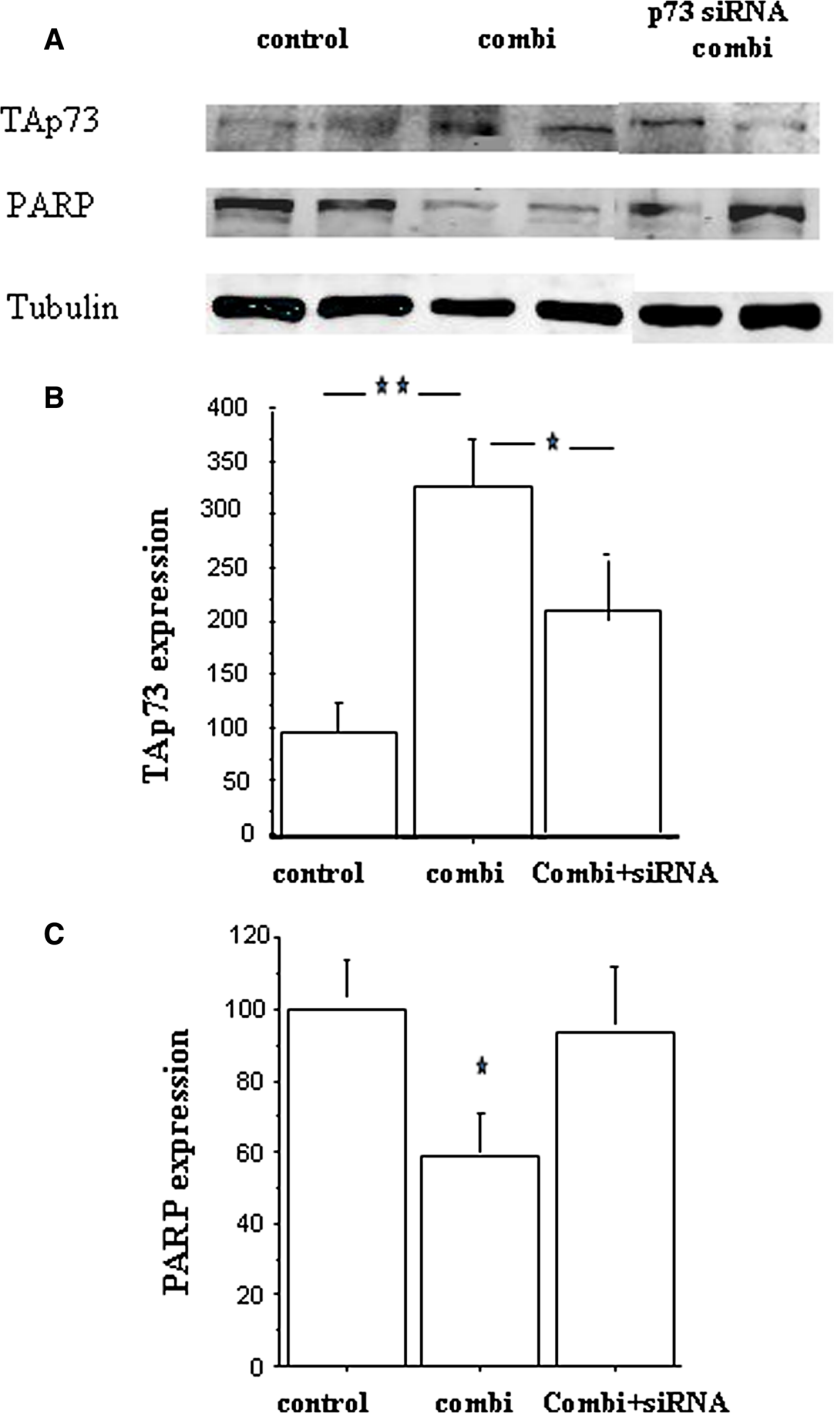
**a** Effect of p73 siRNA pre-treatment on TAp73 and PARP expressions in TT cells receiving 25 μM celecoxib plus 100 μM GTN (combi) during 2 days and 8 h. **b** Effect of p73 siRNA pre-treatment on TAp73 expression in the same TT cells. **c** Effect of p73 siRNA pre-treatment on full length PARP expression in these cells. Graphs obtained from associated results of two experiments on TT cells. For each category, *n* = 6 to 4. Proteins from TT cell lysates were separated by SDS-PAGE using 10 % acrylamide gel and transferred onto nitro cellulose membranes. Immunoblots were probed with specific antibody and a α-tubulin (internal control) antibody. * = *P* < 0.05, ** = *P* < 0.01 (Fisher tests)

Celecoxib/GTN combination induced a strong and significant increase of TAp73 expression in TT cells, after 2 days and 8 h of treatment (*P* < 0.01, Fig. 4a and b). Transfection of siRNA targeting TAp73 isoforms before this treatment, led to a significantly lower level of TAp73 (around minus 50 %, *P* = 0.05). We also observed a decrease of PARP heavy chain expression after the NSAID/GTN incubation (*P* < 0.05, Fig. 4a and c), while TAp73 siRNA suppressed this phenomenon. Thus, as suggested by the previously described correlation, the strong increase in p73 level must be responsible for PARP cleavage and cell apoptosis.

### Confirmation of anti-proliferative effect of the indomethacin/GTN association on rMTC 6–23 cell cultures

**Fig. 5:**
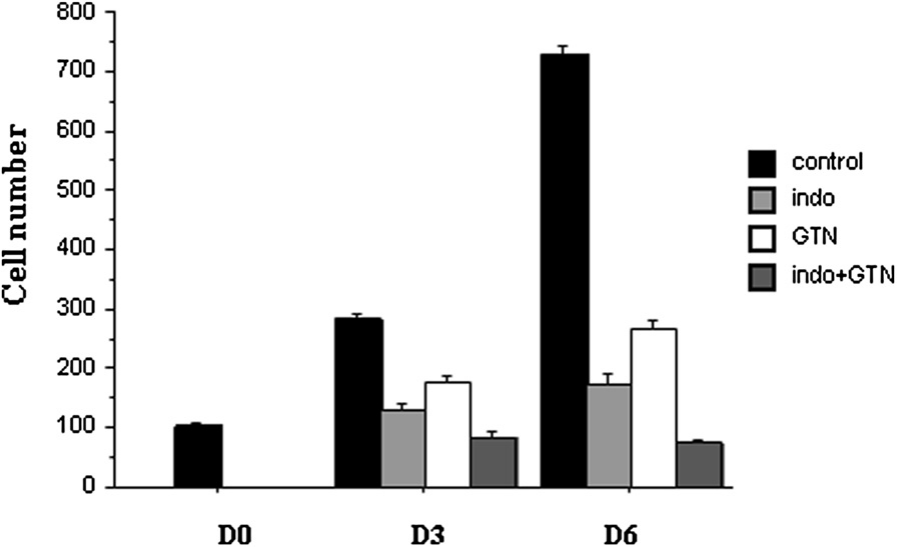
Evolution of the number of viable, control or treated rMTC 6–23 cells: 200 μM indomethacin (indo), 100 μM GTN, or the combination of indomethacin plus GTN, during 6 days. Experiment performed in triplicates. Adherent cells were dissociated with trypsin-EDTA and their number was evaluated with a cell counter (blue trypan exclusion). Drug combination amplified the anti-proliferative effect of each drug (*P* < 0.01 versus indomethacin alone, *P* < 0.0001 versus GTN alone, at D6) and reduced the cell number at D6 versus D0 (*P* < 0.05, Fisher tests)

Anti-proliferative actions of NO and indomethacin were also assessed by incubations with indomethacin alone (200 μM), GTN (100 μM) alone, or combinations of the two drugs in a second MTC cell line (Fig. 5). As observed in TT cells, indomethacine or GTN alone slowed the growth of rMTC 6–23 cell cultures; the combination amplified the anti-proliferative effect of each drug (*P* < 0.01 versus indomethacin alone, *P* < 0.0001 versus GTN alone, at D6) and even reduced the cell number at D6 versus D0 (*P* < 0.05). The apoptosis percentage was low, about 5 % in controls according to Annexin V staining and FACS analysis. Indomethacin did not produced significant change in apoptosis level versus control while GTN alone induced only a low elevation (145 %, *P* < 0.05) but the combination of both led to a stronger increase (308 %, *P* < 0.001), at D6. This ampification was significant (*P* < 0.01, combined treatment versus GTN incubation). Thus, also in this cell line, the strong anti-proliferative action of indomethacin/GTN association resulted from the promotion of apoptotic cell death.

### Anti-tumoral action of combined administration of GTN plus indomethacin on established subcutaneous TT tumors in *nude* mice

**Fig. 6:**
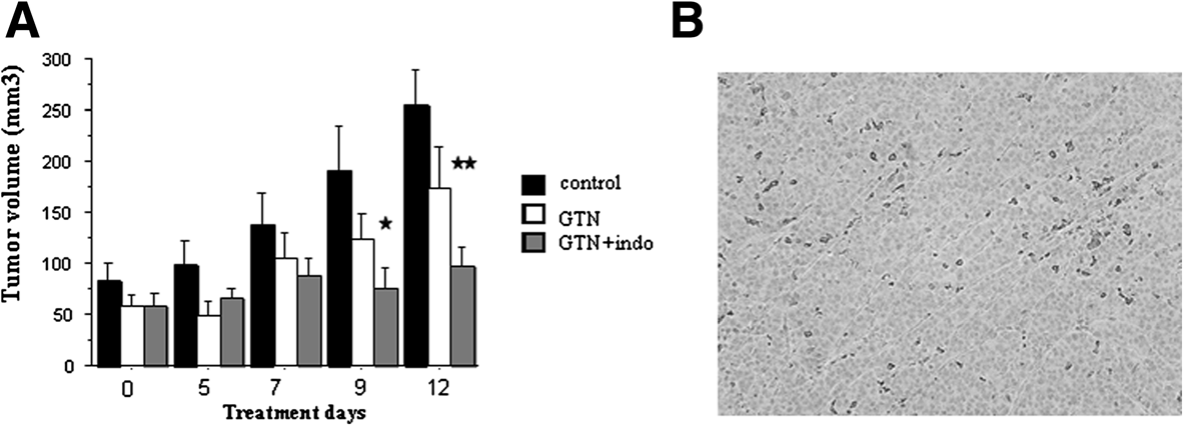
*In vivo* effect of GTN plus indomethacin association on growth of TT xenografts in *nude* mice. Indomethacin was administrated in drinking water (2 mg/kg of body weight × day). Mice treated by GTN received Nitronal sc injections each two days (100 μl during the first week and 150 μl/20 g body weight the second week of treatment). **a** Effect on tumor volumes. The GTN-indomethacin/GTN association stopped the growth of TT tumors from D7 to D12. Significant differences were revealed between tumor volumes by ANOVAs (*P* < 0.0001 combination group versus control, *P* < 0.05 combination group versus GTN treated group; control group, *n* = 5, GTN treated group, *n* = 5 and indomethacin/GTN association, *n* = 4). * = *P* < 0.05, ** = *P* < 0.01 (Fisher tests versus control at D9 or D12). **b** A microphotography of immunostaining of caspase 3 protein revealed numerous apoptotic cells (dark dots) in indomethacin/GTN treated tumors. No staining was observed in the two other groups of tumor

Next, the *in vivo* activity of GTN alone or in combination with indomethacin was determined in *nude* mice. Figure 6 illustrated the effect of treatments on TT xenograft volumes. Subcutaneous injections of GTN did not significantly modified the growth of the subcutaneous tumors while the bi-therapy first slowed it down and then, stopped it from D7 to D12 (global analyses showed a significant effect of the bi-therapy versus control, *P* < 0.0001 and a significant effect of the combination versus GTN alone group, *P* < 0.05). Action on tumor weights at D14 was strictly comparable to effect on tumor volumes at D12 (data not shown).

A HPS coloration of slides showed that control TT tumors were rather only composed of tumoral tissue. The GTN-treated tumors had the same appearance. On the contrary, numerous spaces empty of cancerous cells were visualized in tumors treated by the drug combination. The cavity area represented 19 ± 2.8 % of the total tumor surface. In agreement with these observations, we found numerous cleaved caspase-3 stained cells in these tumors indicating that the combination led to cell death, probably by apoptosis. Such immunostaining observations were rare in the GTN-treated and control groups. The tumor growth arrest with formation of cavities and presence of apoptotic cells suggested that the combined treatment reduced the cancer extension after D7 while GTN alone had no significant effect.

## Discussion

In the present study we demonstrated the anti-proliferative value of NO donor plus NSAIDs on a human MTC cell line. The bi-therapies, celecoxib/GTN, indomethacin/GTN and NO-indomethacin amplified the anti-proliferative effects of each drug alone against TT cells. Indomethacin/GTN combination had the same action on the growth of rMTC 6–23 cells. Since the enhanced cytotoxicity of bi-therapies was correlated with increased expression of TAp73 in TT cells, we proposed that TAp73 might be implicated in the cytotoxic mechanism. In support of this hypothesis, knocking down TAp73 reduced PARP cleavage, a marker of apoptotic cell death. Interestingly, the indomethacin/GTN combination also produced a reduction of tumoral tissue extension and induced cell death, in the *in vivo* model. We have previously reported that the administration of indomethacin alone at the same dose only reduced TT xenograft growth [9].

It has been widely shown that NSAIDs and aspirin prevented the growth of numerous cancers and notably human cancers. The anti-tumor and pro-apoptotic potentials of NO have often been reported. During the last few years, authors have tested various conjugated molecules associating a NO-donor with a non-selective COX inhibitor, *i.e.* classical NSAID (NO-NSAID). They described that NO-NSAID have better anti-proliferative activity than the parent NSAID, *in vitro* and *in vivo*, and in particular, in colon, bladder and prostate cancer [23–26, 13]. Moreover, the administration of NO reduces the side effects of NSAID. Recently, the anti-proliferative action of nitro-oxy derivatives of the COX-2 selective inhibitor, celecoxib, has been assessed in various cell lines. These interesting derivatives of celecoxib which have less toxicity than the parent NSAID, showed an anti-tumor activity comparable to that of celecoxib [27, 28].

With respect to MTCs, our group has found that celecoxib at a low dose and the classical NSAID, indomethacin, reduced the development of tumors arising -from human TT cell injection in *nude* mice [10, 9]. Tomoda et al. [29] also reported that indomethacin have a strong anti-proliferative action on TT cells and two other MTC lines, due to mitotic division reduction without amplification of cell death level. Moreover, we have observed that three MTC cell lines were very sensitive to the anti-proliferative effect of a NO-donor [11]. The present publication reports that a pharmacological dose of GTN, a NO pro-drug used in cardiology, reduced significantly TT cell proliferation *in vitro* via mitotic division decrease. As previously described for other cancer cells, incubation with NO and indomethacin strongly amplified the anti-tumoral effect of treatments alone in TT cell cultures. NO-indomethacin (NCX 530) had a stronger action than the individual NSAID. Interestingly, in our model, the association of GTN with celecoxib also increased the anti-proliferative activity of the drugs used as single agents. After analyses of the mitotic division and apoptosis markers, PCNA and PARP, we found that this phenomenon resulted from the induction of TT apoptotic cell death by combinations of a NO donor with each NSAID, while each molecule alone only acted on mitotic division.

In cancer biology, both positive and negative actions of NO have been reported. Low doses could promote cancer proliferation. Elevation of NO concentration can cause cell damage and apoptosis [15] and the tumor suppressor protein p53 have been implicated in the onset of cell death [16]. Recently, Tebbi et al. [20] described that NO induced the overexpression of the tumor suppressor isoform TAp73α in leukemia cells. In our model, the NO donor GTN, at low dose, did not increase p53 expression and only induced mitotic division reduction without apoptosis induction. This treatment moderately elevated TAp73α expression in TT cells but only after three days of exposition. The pathway leading to *in vitro* proliferation reduction remains unknown. Anyway, only a moderate, not significant growth reduction was obtained *in vivo*. This result could come from the difficulties to reveal a significant effect *in vivo* compared to *in vitro* experiment, from lower NO doses in tissues but also from the promotion of tumor angiogenesis which favors tumor cell proliferation.

The mechanisms of NSAID action are not completely elucidated. The anti-tumor effect of these drugs can result from the decrease of PG by inhibition of synthesis enzymes, COX 1 and/or 2. However, various mechanisms, independent of PG and COX have been also described [30, 31]. Increase of p53 expression was frequently observed after NSAID treatments of various models. In particular, Lau et al. [32] found that COX inhibitors induce apoptosis by increasing p53 stability and nuclear accumulation. Targeting p53 in MTCs may represent an attractive strategy since this protein is not mutated in these tumors [33]. In the present study, we found that only indomethacin increased p53 expression, in TT cells. Indomethacin alone acts on mitotic division [9]. However, p53 level elevation did not seem to intervene in the cell division reduction as this phenomenon appeared before p53 level increase.

Recently for the first time, Lau et al. [34] demonstrated the implication of p73 in the anti-tumor effect of a NSAID: the apoptotic response to celecoxib resulted from the increase of the TAp73β:ΔΝp73 ratio in neuroblastoma cells. In our hand, both celecoxib and indomethacin increased the expression of the TAp73α isoform in TT cells. It is noteworthy that ΔΝp73 and TAp73β were not detected in this cell line. The moderate elevation of TAp73α expression by a 1.5 factor did not promote the anti-mitotic action of the NSAIDs since this anti-tumor effect appeared during the first day of treatment while the variations in protein expression was only observed after more than two days. However we have not investigated post-translational modifications of TAp73α, such as phosphorylation that might be implicated in the anti-mitotic effects of these NSAIDs. Moreover, in our model, the increase of TAp73α after incubation with NSAIDs alone did not induce cell apoptosis. In fact, TAp73α would be a less potent apoptosis inducer than TAp73β [35].

Combinations of NO with one of the NSAIDs did not reinforce the cell division reduction as PCNA level in cells treated with only one simple drug was comparable to the protein expression in cells which have received combined treatment. But interestingly and for the first time, we observed the addition of NO and NSAID effects on TAp73α expression. Thus combinations led to a stronger elevation of this isoform level. Bi-therapies only promoted apoptosis resulting from the TAp73α expression increase as demonstrated by full length PARP western blot after TAp73α siRNA adjunction in TT cell cultures.

In the *in vitro* experiments, we used celecoxib and indomethacin doses that reproduced anti-tumor effects obtained for long term *in vivo* treatments; the NO-donor GTN was used at a pharmacological dose. Thus, the *in vitro* observations described here allow to study a phenomenon that can be also induced *in vivo* as demonstrated in our *in vivo* experiments. More *in vivo* validation has to be done but the bases are set to expect similar results. The expression of p73 must also be verified in human MTCs.

It could be of interest for many reasons to consider clinical studies with NSAID and/or NO-donor therapies. First, these drugs have a low cost and are currently used with many years of experience in healthy subjects. Second, these drugs could be used for a long period at all stage of the disease (advanced or not). Third, combined anti-proliferative actions of NO donor and NSAIDs demonstrated in this study seem independent of known actions of TKI and synergic actions with them could be of value [36]. Unfortunately, clinical experience with use of indomethacin in patients with recurrent or metastatic MTC has been limited to three cases. In only two out of three patients, indomethacin therapy for 3 or 4 months caused marked reduction in tumor mass as well as calcitonin levels [37]. The efficacy of these drugs remains to be determined by clinical trials.
